# Graphene Oxide (GO)-Based Bioink with Enhanced 3D Printability and Mechanical Properties for Tissue Engineering Applications

**DOI:** 10.3390/nano14090760

**Published:** 2024-04-26

**Authors:** Katarzyna Kosowska, Paulina Korycka, Kamila Jankowska-Snopkiewicz, Joanna Gierałtowska, Milena Czajka, Katarzyna Florys-Jankowska, Magdalena Dec, Agnieszka Romanik-Chruścielewska, Maciej Małecki, Kinga Westphal, Michał Wszoła, Marta Klak

**Affiliations:** 1 Foundation of Research and Science Development, 01-793 Warsaw, Poland; korycka.paulina93@gmail.com (P.K.); kamila.jankowska2106@gmail.com (K.J.-S.); asjagiello@gmail.com (J.G.); milena.czajka@fundacjabirn.pl (M.C.); katarzyna.florys.jankowska@fundacjabirn.pl (K.F.-J.); magdalena.dec@fundacjabirn.pl (M.D.); agaromanik@wp.pl (A.R.-C.); kinga.westphal@gmail.com (K.W.); michal.wszola@fundacjabirn.pl (M.W.); 2 Polbionica Sp. z o.o., 01-793 Warsaw, Poland; 3 Department of Applied Pharmacy, Faculty of Pharmacy, Medical University of Warsaw, 1 Banacha Street, 02-097 Warsaw, Poland; maciej.malecki@wum.edu.pl; 4 Laboratory of Gene Therapy, Faculty of Pharmacy, Medical University of Warsaw, 1 Banacha Street, 02-097 Warsaw, Poland; 5 Center for Alzheimer’s and Neurodegenerative Diseases, Peter O’Donnell Jr. Brain Institute, University of Texas Southwestern Medical Center, 6124 Harry Hines Blvd., Dallas, TX 75390, USA; 6 Medispace Medical Centre, 01-044 Warsaw, Poland

**Keywords:** bioink, graphene oxide, extracellular matrix, bioprinting, tissue engineering

## Abstract

Currently, a major challenge in material engineering is to develop a cell-safe biomaterial with significant utility in processing technology such as 3D bioprinting. The main goal of this work was to optimize the composition of a new graphene oxide (GO)-based bioink containing additional extracellular matrix (ECM) with unique properties that may find application in 3D bioprinting of biomimetic scaffolds. The experimental work evaluated functional properties such as viscosity and complex modulus, printability, mechanical strength, elasticity, degradation and absorbability, as well as biological properties such as cytotoxicity and cell response after exposure to a biomaterial. The findings demonstrated that the inclusion of GO had no substantial impact on the rheological properties and printability, but it did enhance the mechanical properties. This enhancement is crucial for the advancement of 3D scaffolds that are resilient to deformation and promote their utilization in tissue engineering investigations. Furthermore, GO-based hydrogels exhibited much greater swelling, absorbability and degradation compared to non-GO-based bioink. Additionally, these biomaterials showed lower cytotoxicity. Due to its properties, it is recommended to use bioink containing GO for bioprinting functional tissue models with the vascular system, e.g., for testing drugs or hard tissue models.

## 1. Introduction

Modern tissue engineering has made tremendous progress in developing hybrid cell culture scaffolds that replicate the extracellular matrix (ECM). The design and manufacture of functional tissues using biomaterials is at the same time the greatest challenge and limitation in the processing and clinical application of artificial constructs. In regenerative medicine, the main goal is not to create a fully functional artificial tissue but to develop an artificial scaffold for culturing cells that mimics their native environment and stimulates their regeneration. The ECM matrix is usually the most important part of artificial constructs. It is made up of a complex network of structural and regulatory proteins arranged in a fibrous matrix that has specific biological functions [1,2,3,4]. The designed scaffold that can replace the native ECM structure should maintain the 3D structure of the cells and allow the diffusion of nutrients, metabolites and soluble factors to ensure tissue regeneration [4,5,6]. During the process of designing and manufacturing artificial scaffolds, it is necessary to take into consideration many features, such as their morphology, porosity, mechanical parameters, surface topography, stability, degradation and biocompatibility [7,8,9,10,11]. The development of new biomaterials is of growing interest to many research groups. Among them, hydrogels containing alginate, gelatin or hyaluronic acid have great potential in bioprinting [12,13,14], regenerative medicine [15,16] and drug delivery systems [17,18]. Bioprinting is an additive manufacturing method using hydrogel biocomposites [19] Hydrogels, as hydrophilic polymeric materials, are able to efficiently retain water, which does not have a negative impact on their structural and physicochemical properties [20,21,22].

An important element in the development of a new biomaterial composition is to give it suitable characteristics that meet technical and biological requirements. It is worth paying attention to such material properties as printability, viscosity, degradability and functionality, as well as biocompatibility, cytotoxicity and bioactivity [23,24]. The composition of an ideal biomaterial should replicate the natural environment for cells as closely as possible. The selection of a material biocomposition with appropriate properties is crucial for the formation of scar structures that can promote cell growth. The selected materials should exhibit properties suitable for use in the chosen bioprinting technique, i.e., a material with good viscosity, adequate stiffness and a degree of cross-linking to achieve stable fibers in precision printing [25,26,27,28,29,30]. Due to hydrogels’ unique characteristics, these biomaterials are suitable for the development of 3D scaffolds [31]. Additionally, to enhance their functional qualities and optimize their usefulness in tissue engineering, the final formulations can be enriched with additional functional ingredients or polymer composites [32]. The most common substances added to organic hydrogels are silica [33], hydroxyapatite [34], gold and silver nanoparticles [35]. Despite the many valuable advantages of using inorganic particles in the bioprinting process, many disadvantages have also been identified, including (i) an increase in shear stress during printing, which can inhibit cell proliferation and viability and affect cell functions; (ii) a decrease in the diffusion of cross-linking agents and (iii) the low homogeneity of the material that prevents the cross-linking process [29,36]. One of the common additives used in modern tissue engineering is graphene oxide (GO) [37], which is an atomically thin sheet with a large surface area and numerous hydrophilic functional groups (e.g., hydroxyl, epoxy and carbonyl). These functional groups make it possible to carry out a wide range of chemical modifications [38]. Its unique properties lead to a number of common applications, including in the design of optical devices [39], photoelectric [40] and metadevices [41]. Recently, biomaterials for tissue engineering have frequently used graphene oxide as a component. The results of studies conducted on 2D cultures determined that the GO improved (i) the adhesion and proliferation of human neural stem cells (hNSCs) [42], (ii) cell growth [43,44,45] and (iii) osteogenic differentiation [45]. Additionally, in 3D studies, the GO was incorporated into alginate- [32,46] collagen- [47] and gelatin methacrylate (GelMA)-based [48] hydrogel scaffolds, resulting in the formation of a functional hybrid scaffold with significant potential for cell differentiation and proliferation.

The application of GO as an additive to biomaterials in biomedicine has not yet been tested and its influence on the immune system is not fully understood. Regardless of the type of biomaterial, any new material that is introduced into the body has an impact on the immune system. The immune response is a complex process that aims to protect against foreign molecules, pathogens or other substances [49]. The incorporation of a biomaterial into a living organism can induce an immune response, as the immune system can recognize this new material as foreign and potentially damaging. This might result in a variety of effects, including inflammation at the site of implantation or incubation with the materials, activation of various types of immune cells and cytokine production [50]. Similarly, the GO-based biomaterial may induce various biological consequences, which depend on a wide range of factors, such as GO properties, concentration, additional GO surface modification, exposure time and individual cell characteristics [51,52]. It is crucial to highlight that research on nanoparticle-based biomaterials is still at an early stage. To determine the clinical utility of new biomaterials and understand their influence on living organisms, systematic studies are crucial in order to avoid and/or inhibit potential adverse immune reactions. The impact of GO-based biomaterials on the cellular immune system also requires detailed investigation [49,51].

The aim of this work was to optimize the composition of a new GO-based biomaterial with unique properties suitable for the bioprinting of functional cell culture scaffolds. The experimental work included the evaluation of properties such as rheology, printability, mechanical strength, elasticity, degradation, soaking and swelling, as well as biological properties, such as cytotoxicity of biomaterials, cell proliferation on the biomaterials, the assessment of lactate dehydrogenase (LDH) and the expression of immune response associated genes (responsible for the expression of cell surface receptors; stress response; oxidoreductases; proteases; transcription factors; signal transduction; cytokines and cytokine receptors; chemokines and chemokine receptors; and cell cycle and protein kinases). For these purposes, we employed several analytical and biological techniques and characterized the selected GO- and non-GO-based biomaterials.

## 2. Materials and Methods

### 2.1. Biomaterials

The bioinks were prepared as a composition of two main components: (i) dense, of a paste-like consistency dECM (decellularized pancreatic extracellular matrix) biomaterial supplemented with an additional amount of dECM powder (Printiss^®^ dECM-PAN; Polbionica Sp. z o.o., Warszawa, Poland); (ii) GO-based hydrogel consisting of a mixture of methacrylated gelatin (TINTBIONIC GELMA 80; Polbionica Sp. z o.o., Poland), methacrylated hyaluronic acid (TINTBIONIC HAMA; Polbionica Sp. z o.o., Poland), GO 2–5 Layer, Dia: 4.5 µm, SA: 420 m^2^/g (Nanografi, Jena, Germany) and glycerol (Chempur, Piekary Śląskie, Poland).

A paste-like dECM biomaterial was obtained as follows: dECM was digested in a pepsin solution (1 mg/mL in 0.01 M HCl; Sigma-Aldrich, St. Louis, MO, USA, Merck Millipore, Burlington, MA, USA) and then neutralized using 0.1 M NaOH (Sigma-Aldrich, USA). After neutralization, an additional amount of dECM powder was added to prepare a paste-like dECM biomaterial.

The GO-based hydrogel was prepared by mixing dissolved GelMa, HaMa, glycerol, LAP and GO in PBS. To prepare the final composition of the bioink, the particular GO-based hydrogel was mixed with a paste-like dECM biomaterial (volume ratio: 1:1). The concentrations of individual components are listed in Table 1. Lithium phenyl-2,4,6-trimethylbenzoylphosphinate (LAP 1.85 mg/mL; Polbionica Sp. z o.o., Poland) was used as the photoinitiator.

### 2.2. Cell Line

The mouse fibroblast cell line L-929 (ATCC^®^, CCL-1TM, Manassas, VA, USA) was used in the studies. The cell line was maintained in Dulbecco’s Modified Eagle Medium (ATCC^®^, Manassas, VA, USA) supplemented with 10% fetal bovine serum (Sigma-Aldrich^®^, St. Louis, MO, USA) and the penicillin–streptomycin solution (Corning™, Glendale, AZ, USA). Cells were maintained at 37 °C in a humidified 5% CO_2_ atmosphere.

### 2.3. Rheology

Rheological analysis of hydrogels and bioinks was performed using the Anton Paar MCR 72 rheometer (Anton Paar, Graz, Austria) with a plate and conical sensor system. A small volume of biomaterial was placed on the sample table and the viscosity was tested at a shear rate of 100 s^−1^ at 25 °C. The storage modulus and the loss modulus of the biomaterials were tested under 1–100% deformation conditions at 14 °C (for hydrogels) and 20 °C (for bioinks), i.e., below the experimentally determined gelation temperature of the materials. All the tests performed for bioinks were carried out using a plate (d = 25 mm) and the table with samples was set at a distance of 1 mm from the plate, while for hydrogels the process was carried out with a cone (d = 50 mm) and the table with the samples was set at a distance of 0.102 mm from the cone.

### 2.4. Printability

**Figure 1:**
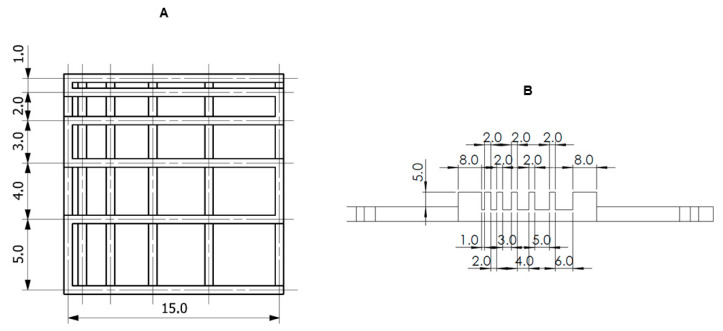
The models of printable structures to evaluate printability in (**A**) fiber fusion test and (**B**) fiber collapse test.

The printability of the developed biomaterials was tested using a three-stage assessment system: a fusion test of fibers printed in the form of a template, a collapse test of a fiber printed on a three-dimensional platform and an assessment of fiber continuity during continuous bioink printing in a volume of 3 mL. Prints were made using a BIO X™ printer (Cellink, Göteborg, Sweden). Figure 1 shows the template and platform model.

#### 2.4.1. Fiber Splicing Test

In order to carry out the fiber splicing test, a g-code was designed, according to which two layers were printed one after the other using the particular material [53,54,55]. The prepared print follows a 0°–90° pattern, which captures the 2D effect and increases the distance between fibers (FD). The distance between fibers was in the range of 1–5 mm with increments of 1 mm. The printing speed, needle diameter and printing distance used in the test were as follows: 20 mm/s, 0.609 mm or 0.437 mm and 0.8 mm. During the test, the material was dispensed at the appropriate range of pressures and temperatures. The printed construct was cross-linked with an external UV-Vis 405 nm lamp for 15 s at 13 W/cm^2^ (Polbionica Sp. z o.o., Poland). Images of the printouts were taken immediately after their fabrication. The images were developed using Carl Zeiss Vision AxioVision Viewer 4.8 software (Carl Zeiss Vision GmbH, Warszawa, Poland). Two parameters described by Equations (1) and (2) were determined from the results, i.e., the percentage of diffusion rate (spreading rate) (*Dfr*) and printability (*Pr*). The pore diffusion rate without material spreading is 0 (i.e., *A_t_* = *A_a_*), and for an ideal reproduction of the model, the printability equals 1.

(1) $$Dfr=\frac{A_{t}-A_{a}}{A_{t}}\cdot 100\%$$

(2) $$Pr=\frac{L^{2}}{16\cdot A_{a}}$$

where *A_t_* is the theoretical pore surface area, *A_a_* is the actual surface area of the pore and *L* is the perimeter of the pore.

#### 2.4.2. Fiber Collapse Test

The deflection at the mid-span of the suspended fiber was analyzed to determine the collapse of the material. For the experiment, a special platform consisting of seven pillars was designed and printed. The particular pillars are spaced from each other by 1, 2, 3, 4, 5 and 6 mm. The dimensions of the two corner pillars are 5 × 10 × 6 mm^3^, while the other five pillars are 2 × 10 × 6 mm^3^. A single fiber of the test material was deposited on the platform, and immediately after that, a picture of the fiber was taken. During the printing process, temperature and pressure conditions were adjusted to the given material, and the print was performed at a speed of 20 mm/s with a 21 G needle (0.609 mm). The collapse area factor (*Cf*), which is the percentage of the actual area after the deflection of the suspended fiber in relation to the theoretical area, was calculated using Equation (3):

(3) $$Cf=\frac{A_{a}^{c}}{A_{t}^{c}}\cdot 100\%$$

where $A_{a}^{c}$ is the actual area under the curve and $A_{t}^{c}$ is the theoretical area under the curve.

If the material is too viscous and is unable to form a “bridge” between two pillars, the actual area is zero and the collapse factor equals 0. On the other hand, if the fiber does not collapse and forms a straight link between pillars, then $A_{t}^{c}$ = $A_{a}^{c}$ and the factor is 100%.

#### 2.4.3. Smoothness and Fiber Continuity

The continuity of the fiber when printing 2–3 mL of the tested bioink was determined using a 0/1 system, where 0 means the fiber is broken and 1 means the fiber is continuous.

### 2.5. Mechanical Testing

The mechanical compressive strength of the printed constructs was investigated using a static compression test. For the experiment, cylindrical specimens (d = 10 mm and h = 5 mm; 100% filling; cross-linking with an external UV-Vis lamp after each layer) were printed using a BIO X™ printer (Cellink, Sweden). Each printed construct was subjected to an external force between 0 and 0.05 N and compressed at a constant rate of 10 mm/min at room temperature until 80% strain was achieved. Measuring points were collected every 0.025 s. After the measurement, a picture of the deformed sample was taken using a camera. Based on the results, the mechanical strength of the samples was calculated as the maximum stress (the ratio of the force to the area of the printed sample) and Young’s modulus as the directional coefficient of the simple stress–strain relationship of the sample in the strain range of 0.1–0.5. An important parameter is also the conventional elastic limit, which is the stress required to deform the samples by 10%.

### 2.6. Degradation

Degradation studies were performed in simulated body fluid (SBF) with or without enzyme (0.1 mg/mL collagenase). Collagenase breaks peptide bonds in collagen, which remains the main component of hydrogels and bioinks. 300 µL of bioinks were placed on Petri dishes and then cross-linked with UV light (λ = 365 nm, 13 mW/cm^2^ for 15 s). The samples were flooded with the appropriate SBF solution and then incubated at 37 °C for 21 days. At given time points, the weight loss or gain of the sample was monitored by fluid withdrawal and lyophilization. Time 0 consisted of freeze-dried and weighed samples immediately after cross-linking. The test for each variant was performed in 3 replicates.

The degree of biodegradation was calculated according to the following formula:

(4) $$Mass\;loss=\frac{w_{0}-w_{1}}{w_{0}}\cdot 100\%$$

where *w*_0_ is the dry weight of the sample immediately after pouring and *w*_1_ is the dry weight of the sample after degradation time *t*.

### 2.7. Effective Swelling and Absorbability

Absorbability: Hydrogels were cross-linked as mentioned in Section 2.6 and incubated in deionized water at 37 °C for 24, 48 and 72 h. At the given time points, water was collected, and the sample was weighed (WS). The test for each variant was performed in 3 replicates. The water absorption coefficient of the swollen gel was calculated as follows:

(5) $$Water\;absorption=\frac{W_{s}-W}{W_{0}}\cdot 100\%$$

where *W*_0_ is the weight of the sample for time 0 h and *W_s_* is the weight of the sample after time *t*.

Swelling ratio: Samples were cross-linked under the same conditions as mentioned in Section 2.6, placed in an aqueous environment (deionized water) and stored at room temperature for 24 and 48 h. After this time, water was collected and samples were lyophilized and weighed. At time 0 h, a sample was subjected to lyophilization immediately after cross-linking. The test for each variant was performed in 3 replicates. The degree of swelling was determined as follows:

(6) $$Swelling\;ratio=\frac{M_{s}-M_{0}}{M_{0}}\cdot 100\%$$

where *M*_0_ is the dry weight of the sample for time 0 h and *M_s_* is the dry weight of the sample after time *t*.

### 2.8. Biological Tests

#### 2.8.1. Viability Assay

The viability of the L-929 cell line (ATCC^®^ Manassas, VA, USA) was assessed using an indirect MTT (3-(4,5-dimethylthiazol-2,5-diphenyltetrazolium bromide) colorimetric assay (MedChemExpress, Monmouth Junction, NJ, USA). Extracts of fragmented biomaterials (HGO1, HGO2, BGO1 and BGO2) were prepared by depositing them on inserts for 24 h in a DMEM culture medium. Subsequently, these extracts, along with a positive control (CP, 0.1% Triton/DMEM), were added to cells seeded in 96-well plates at densities of 1 × 10^4^/well, 5 × 10^3^/well and 2.5 × 10^3^/well. The cells were then incubated at 37 °C, 5% CO_2_ in the DMEM medium for 24, 48, and 72 h, respectively. At the end of the incubation period, the medium was removed and 1 mg/mL MTT (Sigma Aldrich^®^, USA) was added. The absorbance of formazan was measured at 570 nm using a microplate scanning spectrophotometer (BioTek, Winooski, VT, USA). Cell viability was calculated in comparison to untreated cell culture (negative control, CN) using the formula

(7) $$Cell\;viability\;\%=\frac{OD\;sample}{OD\;negative\;control}\cdot 100\%$$

where *OD sample* is the absorbance of the sample at λ = 570 nm (average of 5 replicates) and *OD negative control* is the absorbance of the negative control at λ = 570 nm (average of 6 replicates).

According to the current ISO 10993-5:2009(E) norm [56], a material that does not show cytotoxicity is one for which the cell viability is at least 70%

#### 2.8.2. Cytotoxicity Assay

To investigate the direct impact of the biomaterial on the L-929 cell line, cells were seeded into 6-well plates pre-coated with GO-supplemented biomaterials (HGO1, HGO2, BGO1 and BGO2) at densities of 2.5 × 10^5^/well, 6.3 × 10^4^/well and 4 × 10^3^/well. The cells were subsequently incubated at 37 °C and in a humidified 5% CO_2_ atmosphere in a DMEM medium for 1, 3 and 7 days.

The LDH-Glo™ Cytotoxicity Assay (Promega, Madison, WI, USA) was used to evaluate cytotoxicity resulting from cell interactions with biomaterials using the L-929 cell line (ATCC^®^, CCL-1™, Manassas, VA, USA). The assay was conducted at three distinct time points (1, 3, and 7 days).

To carry out the assay, the culture medium was collected and diluted at a ratio of 1:100 in the LDH Storage Buffer. The samples were stored at −20 °C until testing. The entire procedure had been carried out according to the manufacturer’s protocol. The luminescence signal was measured using a microplate reader (BioTek Synergy H1 Plate Reader) after a one-hour incubation at room temperature.

#### 2.8.3. The Evaluation of Cell Proliferation Utilizing the Alamar Blue Assay

The proliferation of the L-929 cell line (ATCC^®^, CCL-1™, Manassas, VA, USA) was measured at 1, 3 and 7 days after being directly cultivated on the surface of biomaterials (HGO1, HGO2, BGO1 and BGO2) using the Alamar Blue assay (Invitrogen™, Waltham, MA, USA). According to our modified protocol, cells were incubated for 24 h with Alamar Blue reagent in a ratio of 1:10. After incubation, 100 µL of the medium was transferred to black plates to absorb light and reduce background and crosstalk. The absorbance of each sample was then measured at 530 nm and 590 nm using a plate reader.

#### 2.8.4. The Assessment of the Expression of Immune Response Genes in the L-929 Cell Line Following Its Interaction with BGO1 Biomaterial

Real-time PCR gene expression analysis was used to assess the fold change expression of immune response-related genes. The L-929 cell line required to assess the expression level of immune function genes was seeded at the density mentioned above, directly on the biomaterial BGO1, which was characterized by improved printability, a lack of variation in rheological properties, increased mechanical strength in comparison to the reference sample (without GO) and a lack of cytotoxicity. Total RNA from the L-929 cells was isolated using the PureLink™ RNA Mini Kit (Invitrogen™, Waltham, MA, USA). The purity of the extracted RNA was assessed using the NanoDrop™ One/OneC Microvolume UV-Vis Spectrophotometer (Thermo Scientific™, Waltham, MA, USA) by measuring absorbance at 260 nm and 280 nm wavelengths. The reverse transcription reaction was performed using the High-Capacity RNA-to-cDNA kit (Applied Biosystems™, Waltham, MA, USA) according to the manufacturer’s instructions. Gene expression was investigated with TaqMan^®^ Array 96-WELL FAST Plates (Applied Biosystems™): mouse immune response (cat no: 4418856; Waltham, MA, USA). All samples were analyzed in duplicates using 50 ng total RNA/sample. Real-time PCR was performed using a CFX96 Touch Real-Time PCR Detection System instrument (Bio-Rad, Hercules, CA, USA). The results were normalized to the *Hprt1* reference gene. The relative gene expression was calculated using the 2^−ΔΔCq^ method [57]. The evaluation of immune response gene expression was conducted in L-929 cells cultured on the BGO1 biomaterial, known for its favorable physicochemical and biological characteristics. The control sample was cells cultured at 37 °C in a humidified 5% CO_2_ atmosphere. The experiments were performed at two time points: after 1 and 7 days of incubation. In this study, 92 assays for immune response-associated genes and 4 assays for candidate endogenous control genes were evaluated.

The STRING database was exploited to prepare the chain graphs depicting the physical and/or functional interactions between the proteins encoded by the investigated transcripts [58].

#### 2.8.5. Statistical Analysis

The results are presented as mean and standard deviation. The Shapiro–Wilk test was used to determine the distribution’s normality. For comparisons among samples in the Alamar Blue assay and LDH assay, a one-way analysis of variance (ANOVA) was employed. Subsequently, the Tukey test, as a post hoc analysis, was conducted to determine statistically significant differences between samples at specific time points. The significance thresholds were * *p* < 0.05, ** *p* < 0.01 and *** *p* < 0.001. The statistical analysis was carried out using Statistica 13.1 software (StatSoft Polska Sp. z o.o., Cracov, Poland).

## 3. Results and Discussion

### 3.1. Rheology

**Figure 2:**
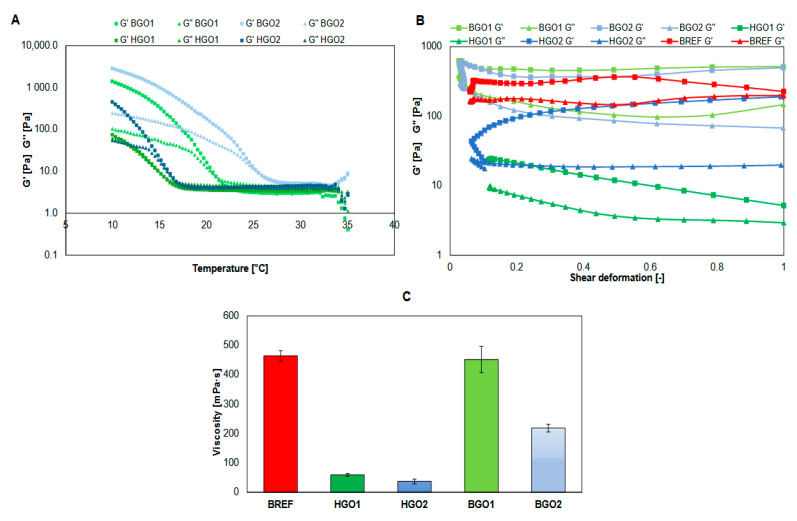
Results of rheology testing, where (**A**) shows gelation point, (**B**) shows complex modulus and (**C**) shows viscosity of biomaterials.

Due to its utility in the 3D-bioprinting process, the biomaterial must be characterized by rheological properties, such as the storage modulus (G′), which refers to the elastic properties and serves as a measure of retaining the elastic shape, as well as the loss modulus (G″), which represents the viscous part or the amount of energy dissipated in the sample [59]. The results revealed that the BGO1, BGO2 and BREF bioinks showed a higher storage modulus (G′) (higher energy storage capability of the material) than the loss modulus (G″) in the tested range of oscillation amplitude (see Figure 2B), which suggests that they can be regarded as mainly elastic biomaterials. This feature is especially important in the context of the use of such biomaterials in the process of bioprinting, as the greater elasticity of the biomaterial allows it to be extruded more easily [60]. The material should exhibit viscoelastic features, so it must show viscous-like behavior for better extrusion and to eliminate issues related to nozzle clogging, and elastic-like behavior to maintain the stability of the printed fiber. Since the storage modulus is a measure of energy that has to be put into the sample to distort it, it has to be emphasized that for bioprinting purposes, the biomaterial’s resistance to deformation cannot be too high as it may prevent its extrusion. As a consequence, the liquid-like properties of the biomaterial cannot outbalance the elastic ones and vice versa; therefore, the appropriate biomaterial should exhibit a balance between the storage and loss moduli [61]. The value of the storage modulus obtained for the reference bioink (BREF a non-GO-BGO1 variant) is slightly lower than the values determined for the bioinks supplemented with GO, which may suggest that the addition of this nanomaterial has an impact on the biomaterial’s elasticity and increases its solid-like behavior. Additionally, differences in the ratios of GelMa and HaMa (BGO1 and BGO2) did not introduce significant changes in the component values of the complex modulus. Chen et al. [62] and Li et al. [60] have proven that low GO concentrations in bioink enable the connection of polymer chains via hydrogen bonding, which increases elastic energy storage capacity (G’). Higher GO concentrations prevent the formation of hydrogen bonds and reduce the biomaterial’s elasticity but do not have any influence on its viscous behavior (G″). However, the lack of a paste-like dECM significantly decreases the complex modulus values: 2 times for HGO2 and 30 times for HGO1, and since the complex modulus is a measure of the material’s overall resistance to deformation, the addition of a paste-like dECM results in an increase in the biomaterial’s solid-like behavior.

Figure 2C shows the viscosity values measured for all the variants (both bioinks and hydrogels). Hydrogels have viscosity values 100 times lower than the corresponding bioinks, while the comparison between the particular variants HGO1/BGO1 vs. HGO2/BGO2 revealed that the highest values were determined for HGO1/BGO1. It might be due to the fact that HGO1/BGO1 variants contain a lower concentration of both HaMa and GelMa. The highest viscosity was determined for BGO1 (approx. 450 mPa·s), while the lowest for HGO2 (approx. 37 mPa·s). It has to be emphasized that the suitable bioink with potential for application in bioprinting should rather exhibit lower viscosity in order not to clog the printhead [63]. However, the bioink viscosity must be adapted to its potential use, since this feature affects not only the printability but also its compatibility with cells. It is commonly known that more viscous bioinks provide stronger mechanical support and are more deformation-resistant, which increases the printing fidelity but, on the other hand, the excessive viscosity significantly reduces the survival of cells. Additionally, higher viscosity requires applying higher printing pressure, which has a negative impact on the survival and functionality of cells. In turn, lower viscosity is more likely to provide suitable conditions for cells but it also negatively affects printing fidelity and resolution [64].

### 3.2. Degradation, Absorbability and Swelling Ratio

**Figure 3:**
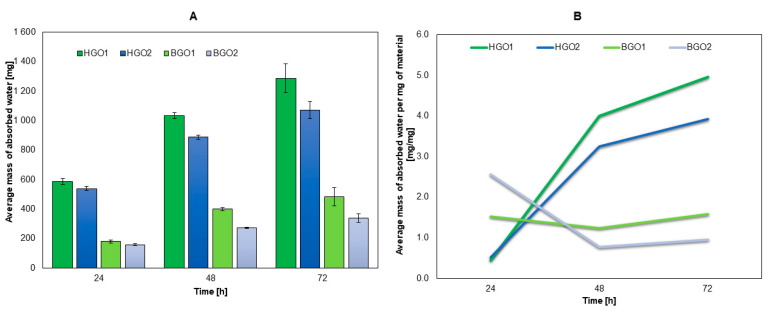
The results for water absorbability: the weight of absorbed water (**A**) and the weight of absorbed water per mg of biomaterial (**B**).

Based on the results (see Figure 3A), it can be seen that significantly more water is absorbed by hydrogels (HGO1 and HGO2) compared to bioinks (BGO1 and BGO2). In general, biomaterials absorb most of the water during the first 48 h and after this time water absorption decreases, which is a consequence of biomaterial saturation.

The analysis of water absorption per mg of sample (Figure 3B) revealed that after 24 h, the hydrogels (HGO1 and HGO2) exhibit the lowest water absorption per mg of sample but after 48 h this trend is reversed. For the bioinks (BGO1 and BGO2), it was determined that the highest absorption occurs after 24 h and it decreases in the following days. It can be concluded that bioinks containing dECM can be easily saturated with water on the first day, after immersion in the liquid.

The degree of hydrogel swelling was 6.5 (HGO2) and 8.5 (HGO1) times higher than for bioinks. This means that the addition of dECM increases the cross-linking densities, which causes a reduced water adsorption capacity. High values of the hydrogel swelling ratio may be due to the content of GelMa, whose swelling ratio for a concentration of 7.5% *w*/*v* is 1273% [65].

The degree of degradation after 21 days was 50–90% for non-enzymatic degradation and 80–90% for enzymatic degradation. The bioinks were more resistant to non-enzymatic degradation than hydrogels (BGO1 < HGO1 and BGO2 < HGO2), while the results of enzymatic degradation revealed that both bioinks and hydrogels had similar values for the degree of degradation (HGO1 and BGO1 as well as HGO2 and BGO2). Since the addition of dECM increases the cross-linking densities, it is believed that it also increases the resistance of the biomaterial to non-enzymatic degradation.

The degree of degradation is very important, especially for biomaterials employed in bioprinting. Degradation should occur at a well-defined rate so that cells can create their own network of extracellular matrix. Both GelMa and HaMa degrade easily. A 10% GelMa (degree of substitution above 70%) degrades completely within 6–8 h in a Hanks’ Balanced Salt Solution environment with the addition of collagenase [66]. On the other hand, a 1% HaMa degrades completely within 5 days in the PBS environment [67]. The obtained results have proven that the higher the cross-linking density (the addition of GO and dECM), the more stable the network and the slower the degradation (Table 2).

### 3.3. Printability

In order to determine the optimal printing parameters, several fibers were printed using BREF, BGO1 and BGO2 bioinks. As can be noticed, the use of GO-based bioinks generates the need to improve printing pressure compared to the reference sample (BREF). For BGO1 and BGO2, the value of applied pressure was about 20 and 30 kPa higher than the values determined for the reference bioink (BREF). To evaluate the optimal printing parameters, one should take into consideration not only the way of manufacturing a 3D object with the desired physicochemical characteristics but also the influence of printing parameters on cell survival. Many experiments are currently being conducted to determine the effect of applied extrusion pressure on cell survival and model mapping [29,68,69]. Since the higher concentration of the additive requires the application of higher printing pressure [70] and this may have a negative impact on cell survival, such bioink will not find application in bioprinting even if it exhibits distinctive physicochemical properties and improved printability. The influence of the additive on printing pressure was confirmed by Chor et al. [32]. The printability tests of an alginate-based material supplemented with GO required the application of much higher pressures. Similar results were obtained for GO-based alginate–gelatin material by Li et al. [60].

The application of extrusion bioprinting requires considering such a parameter as the magnitude of shear stress. This feature depends on the extrusion pressure, nozzle diameter, printing speed and viscosity of the printed material [36]. Shear stress can have a significant impact on cellular processes and can alter cell signaling and protein expression [71]. In general, bioinks should have low viscosities in order to pass through printing nozzles [72]. However, using high-viscosity bioinks and narrow nozzles, it is possible to print 3D objects with higher resolution. Yet, these two features—the high viscosity of the material and the small diameter of the nozzles—generate excessive shear stresses, which has a negative impact on cells that are loaded in the bioink. Additionally, the printing of high-resolution 3D objects takes longer, so it is essential to employ dedicated syringes and advanced motor controls to reduce the time needed [73]. Therefore, it is crucial to maintain a delicate balance between the applied shear stress and the resolution of the 3D object. It is important because excessive shear stress can harm cell membranes and induce cell apoptosis [36].

Another important feature that has to be evaluated is the printing temperature. In our study, the bioink composition did not affect the printing temperature; in each case, it was possible to print a continuous fiber at the temperature range of 24 to 25 °C. It is commonly known that high temperatures are impoverishing for living cells, so methods such as thermal inkjet printing are not suitable for tissue engineering [74]. However, this method can be utilized to print 3D scaffolds using acellular materials. For the BREF material, a smooth and continuous fiber was obtained at a temperature of 24–25 °C and an extrusion pressure of 30–35 kPa, while for the BGO1 material, the optimal printing parameters were a temperature of 25 °C and a pressure of 50–60 kPa, and for BGO2, a temperature of 25 °C and a pressure of 70–75 kPa.

In this study, we also determined the optimal extrusion rate for our biomaterials (20 mm/s). It has to be emphasized that this value depends on the biomaterial’s properties and its composition. In 2022, Zhu et al. [75] found that the bioprinting of an artificial ear using a biomaterial supplemented with other nanoparticles (Cu-doped bioglass nanoparticles) requires maintaining a 3D-printer extruder’s flow rate at 10 mm/s. This suggests that for each kind of biomaterial, it is essential to first optimize the bioprinting conditions, as they might be affected by several factors, such as the properties of the components and/or the method of sample preparation.

**Figure 4:**
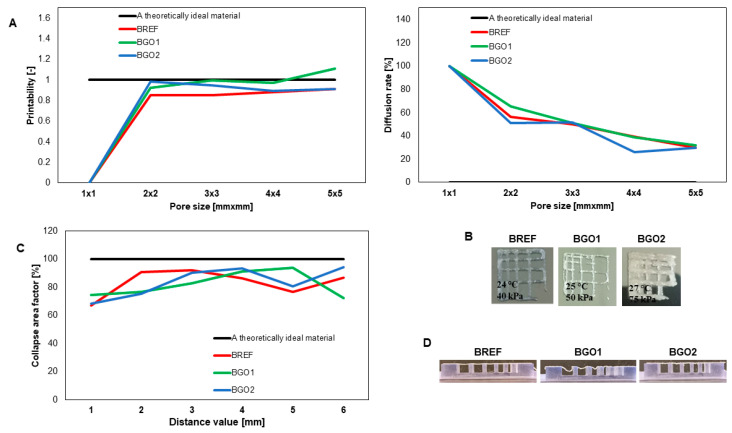
The printability of graphene bioinks. (**A**) The fiber fusion test results along with printing parameters; (**B**) the photographs of prints from the fiber fusion test; (**C**) the fiber collapse test results on the platform—fiber collapse rate; (**D**) the photos of fibers printed on the platform.

Figure 4A shows the results of the confluence of printed fibers. A photographic image of the printed model is shown, along with the printing parameters and the dependence of the material spread rate and printability coefficient on the size of the printed pore. In our study, for all materials, as the pore size increases, the diffusion rate decreases and printability increases for pores larger or equal to 4 mm^2^. The lowest diffusion rate and highest printability for a pore of 4 mm^2^ were determined for the BGO2 material. Figure 4C shows the results of the collapse test of a fiber printed on a special platform. All materials enabled the printing of a stable and continuous fiber; however, a more satisfactory result was obtained when using BGO2 bioink. In each case, printability close to 0.8 was achieved. It has to be emphasized that printability ca. 1 and the material’s relatively low diffusion rate is an important parameter that determines the material’s usefulness in bioprinting [54,76].

In this research area, it is very common to present photographic images of printed models with pore geometries highlighted. Habib et al. [54] determined parameters characterizing printability such as *Pr* and *Dfr* (diffusion rate) for an alginate-based material containing carboxymethylcellulose (CMC). Materials containing a higher concentration of CMC—3 and 4% show a lower diffusion rate and a higher printability than the non-CMC material. In turn, Im et al. [77] determined the confluence of fibers and the printability parameter (*Pr*) for an alginate material supplemented with cellulose nanofibers and polydopamine nanoparticles. It was confirmed that a printability value equals approximately 1, which may suggest that this material is very similar to the ideal one, but it is worth noting that any deviation from this value (1) indicates non-ideal printability. It was also determined that the addition of nanomaterial to free alginate leads to an increase in the *Pr* value, which confirms that this material has properties closer to the ideal state (value 1) [76].

In this study, we have determined that the use of GO has a positive impact on the printability of the biomaterial. However, one should keep in mind that additives may also alter the rheological and biological properties of the biomaterial, which may exclude its application in the bioprinting processes even if its printing characteristics are similar to the parameters of the ideal material [78].

### 3.4. Mechanical Properties

**Figure 5:**
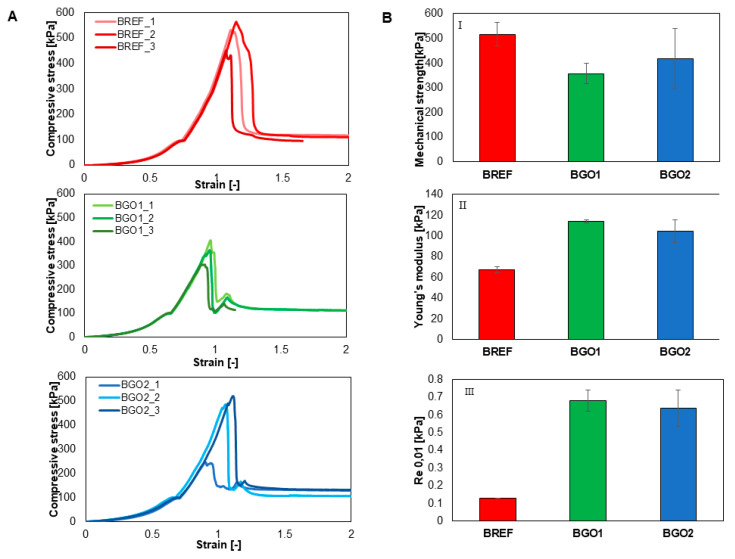
The results from the static compression test. (**A**) Stress–strain behavior. (**B**) Mechanical parameters: I, mechanical strength; II, Young’s modulus; III, elastic limit.

The mechanical parameters of 3D-printed objects were determined using the static compression test to evaluate the sample’s response to crushing. The elastic limit is the stress value that is required to deform 10% of the height of the specimen, while Young’s modulus represents the stiffness of the material and was determined as the directional coefficient of the most rectilinear segment on the stress–strain curve, in the strain range of 0.1–0.5. The results of the static compression test are shown in Figure 5.

In general, the nanomaterial additives should increase the mechanical strength of the printed object [79,80]. In our study, the applied force in each case led to the deformation of a 3D structure, and the GO-based bioinks were characterized by higher values of Young’s modulus and conventional yield strength. However, it has to be emphasized that these bioinks exhibited lower crush resistance than the reference sample. Yet, one should keep in mind that this test is suitable for determining the resistance of the 3D object and not the biomaterials that were used to produce this object [81].

### 3.5. The Assessment of GO-Based Biomaterial on Cell Viability of the L-929 Cell Line

**Figure 6:**
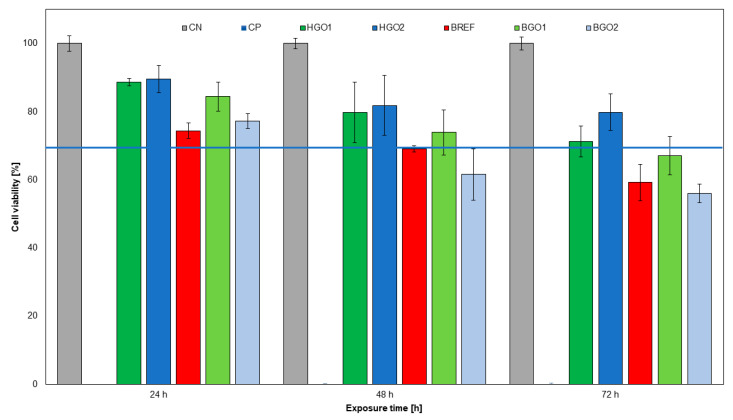
The effect of GO-enhanced biomaterial extracts on L-929 cell line in an indirect MTT assay. The blue line indicates the guidelines established by ISO 10993-5:2009(E), according to which >70% cell viability of the L-929 cell line determines the absence of cytotoxicity of the test biomaterial.

The indirect MTT assay was performed at three time points: 24, 48 and 72 h after the application of extracts derived from the biomaterials. Based on the results, no cytotoxic effect of the tested biomaterial was demonstrated. The viability was converted relative to cells that were not treated and cultured under standard conditions. The lack of cytotoxicity was observed for all tested biomaterials: HGO1 88.6% ± 1, HGO2 89.5% ± 4, BGO1 84.4% ± 4 and BGO2 77.3% ± 2 after 24 h exposure. The reference material was 74.4% ± 2 at this point in time. During the second day of incubation, cell viability gradually began to decrease and was HGO1 79.8% ± 9, HGO2 81.8% ± 9 and BGO1 73.9% ± 7. The cytotoxicity effect increased on the second and third day for BGO2 61.6% ± 8 and BGO1 67.1% ± 6, respectively (Figure 6). 

### 3.6. The Assessment of the L-929 Cell Line after Exposure to the GO-Based Biomaterials

**Figure 7:**
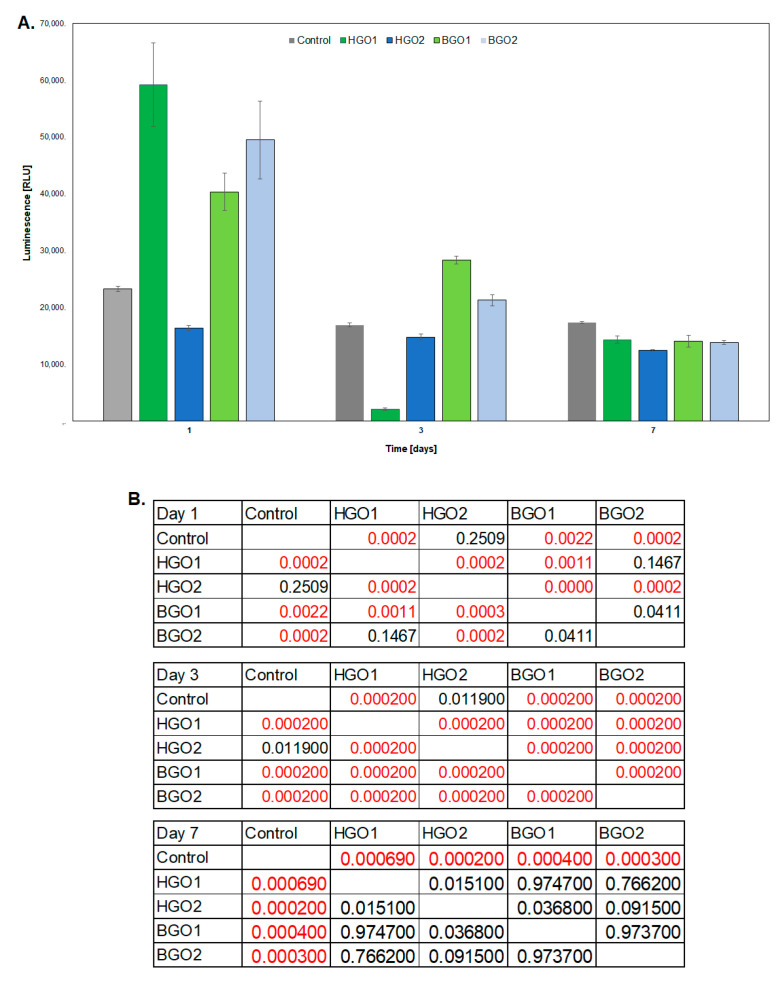
The influence of GO-based biomaterials on the viability of L-929 cell lines. The effect was measured using an LDH assay. (**A**) LDH assay, (**B**) statistical analysis; red: statistically significant differences, black: no statistically significant differences.

It was observed that as the cells were incubated on the biomaterial, the level of luminescence decreased, which indicates the release of lactate dehydrogenase (LDH) (Figure 7A). When the cells were exposed to the HGO1 extract, the luminescence level was 59,168 ± 7386 on the first day. This value decreased to 14,357 ± 594 on the seventh day (** *p* < 0.01). Figure 7 shows that the LDH release for control cells remained relatively constant at the level of 17,000–20,000. The HGO2 biomaterial exhibited luminescence levels comparable to the control cells throughout the incubation period, amounting on average to 15,000. The relative luminescence unit (RLU) values for BGO1 and BGO2 on the first day of incubation were 40,351 ± 3277 and 49,486 ± 6815, respectively, and decreased over time (Figure 7) until the last day of incubation, when they reached values equivalent to those of control cells. On the first day of the LDH assay, no statistically significant differences were noted between the control and HGO2, HGO1 and BGO2, and BGO1 and BGO2. Only the control and HGO2 showed statistically significant differences on the third day. On the seventh day, statistically significant differences were observed solely between the control and BGO1, BGO2, HGO1 and HGO2, respectively. In addition, the LDH assay on the first day showed a lack of statistically significant differences between control and HGO2, HGO1 and BGO2, and BGO1 and BGO2. On the third day, a lack of statistically significant differences was noted between control and HGO2, and on the seventh day, statistically significant differences were only observed for control and BGO1, BGO2, HGO1 and HGO2, respectively (Figure 7B).

**Figure 8:**
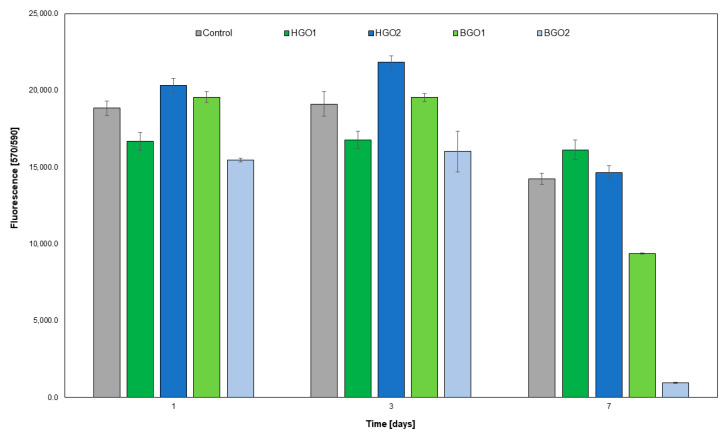
The influence of GO-based biomaterials on the proliferation of L-929 cell lines. The effect was measured using the Alamar Blue assay.

The results of the proliferation rate evaluation, as depicted in Figure 8 indicate that the level of fluorescence remained close to the control cells’ relative fluorescence units (RFU), and was 19,000 for the HGO2 biomaterial across all three time points (RFU 21,000). For the BGO1, RFU was 20,000 on the first and third day of incubation but decreased on the seventh day (up to 9000). For HGO1 and BGO2, the RFU levels were similar to the control cells, on both the first and third day of the experiment (17,000 and 16,000, respectively). However, for these two samples, we detected a significant decrease in fluorescence on the seventh day of incubation (BGO1—9000 and BGO2—1000). All the samples tested with the Alamar Blue assay showed statistically significant differences at a particular time point (Figure 8).

#### Immune Response Genes Expression Levels after L-929 Cell Line Exposure to the BGO1 Biomaterial

**Figure 9:**
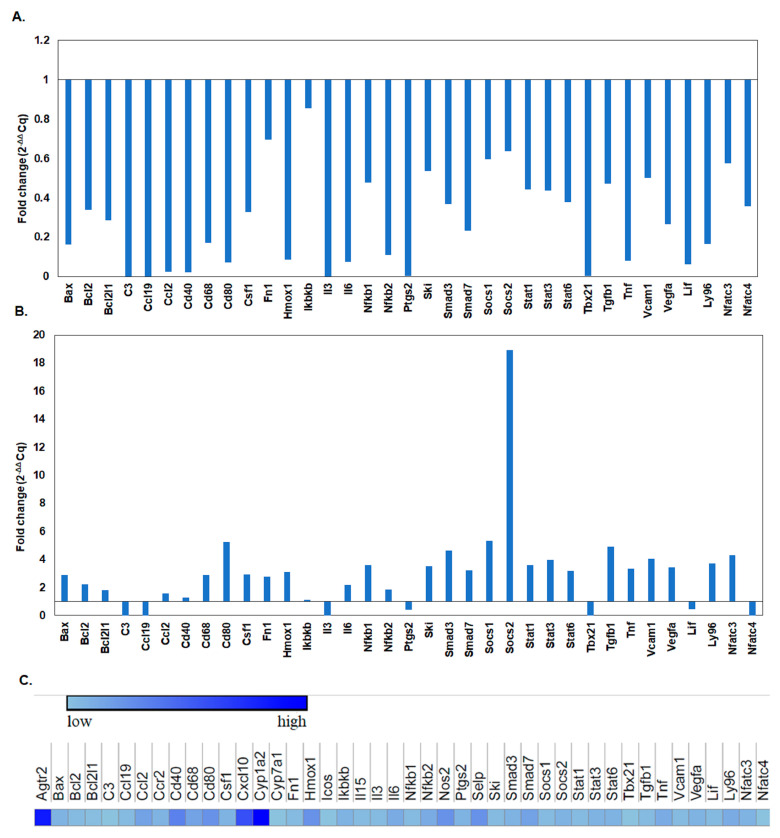
The relative expression of immune response-associated genes in mouse fibroblast cell lines after exposure to the BGO1 biomaterial. This analysis focuses on the measuring of the gene expression on the first day of incubation (**A**) and after seven days of incubation (**B**). Additionally, we assess the fold change in gene expression between the first and seventh day of incubation (**C**) comparing these to the control cells from the first day of incubation. The image was generated using software available at www.software.broadinstitute.org/morpheus/ (accessed on 14 August 2023). Here, lower values correspond to a reduction in gene expression (bright blue color), while higher values indicate an increase in gene expression (dark blue color).

Genes with a Cq value below 35 were selected for inclusion in the analysis. In the initial phase, both control (non-exposed) and exposed L-929 cells were analyzed on the first and seventh day of incubation on the surface of the BGO1 biomaterial (Figure 9A,B). Noteworthy differences in gene expression fold change were observed for several genes (Figure 9A–C). On the first day of incubation, a negative fold change in the expression of most genes compared to control cells was determined (with values < 1); (Figure 9A). Particularly noteworthy were the lowest values (~0, Figure 9A) determined for genes such as *Agtr2*, *C3*, *Ccl19*, *Ccl2*, *Cd40*, *Cxcl10*, *Cyp1a2*, *Fas*, *Il3*, *Nos2*, *Ptgs2* and *Tbx21*. Similarly, genes such as *Ccl5*, *Cd80*, *Hmox1*, *Il6*, *Selp*, *Tnf* and *Lif* exhibited a consistent fold change decrease of approximately 0.07. The *Cyp7a1* gene had a similar expression as the control sample (Figure 9A). Other genes ranged from 0.2 to 0.8-fold change in expression (Figure 9A).

After seven days of incubation on the surface of the BGO1 biomaterial, significant differences in gene expression were also observed, compared to control cells (non-exposed, Figure 9B). Most genes exhibited a fold change increase during this incubation period. The Socs2 gene displayed the highest expression fold change (19—see Figure 9). Genes such as *Cd80*, *Il5*, *Smad3*, *Socs1*, *Tgfb1*, *Vcam1* and *Nfatc3* also showed increased levels, with values of approximately 4–5 (Figure 9B). Conversely, significant negative fold changes (near 0) were observed for genes *C3*, *Ccl19*, *Icos*, *Il3*, *Tbx21* and *Nfatc4*. Additionally, the Ptgs2 (0.41) and Lif (0.47) genes exhibited a decreased expression. Moreover, for genes *Ccl2*, *Cd40* and *Ikbkb* we determined similar levels of expression to those of control cells (non-exposed) at 1.55, 1.29 and 1.11, respectively (Figure 9B).

An analysis based on the fold changes of immune response-associated genes in cells incubated for a given time on the surface of the biomaterial was also performed. In this case, cells cultured on the surface of the BGO1 biomaterial on the first day of incubation served as the control (Figure 9C). The highest fold increase in expression was observed for the *Cyp1a2* gene, as depicted in Figure 9C. The second-highest increase in expression was observed for the *Agtr2* gene. Notable positive fold changes were also observed for the *Cxcl10*, *Cd80*, *Hmox1*, *Cd40*, *Nos2* and *Selp* genes, respectively (Figure 9C). The *Cyp7a1* gene showed a change in expression similar to the results obtained for cells incubated on the BGO1 surface on the first day (Figure 9C). A negative fold change was determined for the *Icos*, *Tbx2* and *Nfatc4* genes (bright blue color, Figure 9C). The remaining genes exhibited a positive fold change in comparison to the control samples on the first day of incubation.

In this part of research, we conducted comprehensive assessments of the cytotoxicity of selected biomaterials, and we evaluated the proliferation of cells exposed to a given biomaterial, as illustrated in Figure 7, Figure 8 and Figure 9. Moreover, we determined the expression profiles of immune response-associated genes in mouse fibroblast cells, as depicted in Figure 9. These investigations required the analysis of 92 genes that play key roles in orchestrating the immune response—a highly intricate defense mechanism according to which cells and proteins provide protection against pathogens. When the antigen enters the body, it is first recognized, and its presence induces the activation of a signaling cascade to overcome infection. This process recruits various components, such as surface receptors, signaling molecules, cytokines, chemokines, etc., to remove deleterious stimuli [82]. The biomaterial that was analyzed contained GO, a single-atomic layered material made by the oxidation of graphite. Based on the findings reported by Mukherjee SP et al., it was determined that various GO derivatives may cause a different response of the immune system [49]. It can be determined using fold-change analysis to determine the expression levels of certain genes involved in the immune response. In GO-based biomaterials, a critical factor is the oxygen level of GO (atomic ratio of carbon to oxygen) [83]. The reduction of oxygen-containing functionalities reduces its hydrophilicity. The presence of GO may have an impact on the hydrophobic/hydrophilic properties of the biomaterial surface. Additionally, the distribution of hydrophilic GO within the biomaterial may have also altered its hydrophilicity. Therefore, we assumed that the alterations in gene expression profiles that we observed might have been induced by the presence of hydrophilic GO. Indeed, these alterations were observed during the incubation of cells on the biomaterial, as illustrated in Figure 9A,B. In some cases, we determined the reduced expression of certain genes, which may confirm that GO does not rapidly induce an immune system response.

The above-mentioned alterations may lead to a reduced secretion of cytokines and diminished activation of immune cells. These changes were also investigated by other research groups: Lategan K. et al. [84], Yang Z. et al. [85] and Cebadero-Dominguez Ó. et al. [86]. As can be seen in Figure 9A, after 1 day of incubation, a decrease in expression of the following genes was observed: *Agtr2*, *C3*, *Ccl19*, *Ccl2*, *Cd40*, *Cxcl10*, *Cyp1a2*, *Fas*, *Il3*, *Nos2*, *Ptgs2*, *Tbx21*, *Ccl5*, *Cd80*, *Hmox1*, *Il6*, *Selp*, *Tnf* and *Lif*. These genes are associated with various biological processes and can mutually influence one another under diverse physiological and pathological conditions. The intricacies of their interactions are context-dependent and influenced by factors such as tissue types, external stimuli and other variables. For example, *Tnf*, a tumor necrosis factor, plays a role in inflammation induction and can impact the expression of *Ccl2*, *Ccl5* and *Il6*, thereby intensifying the inflammatory response [87], as illustrated in Figure 9A. Similarly, *Il6* can modulate *Hmox1* expression in response to oxidative stress and influence TBX21 activation in Th17 T cells [88]. On the other hand, CCL2 and CCL5 participate in the recruitment of inflammatory cells [89] and can be regulated by Il6, Tnf and other inflammatory mediators. PTGS2 is involved in metabolism and can be influenced by various factors, including inflammatory cytokines. CD40 regulates the expression of Cd80 on antigen-presenting cells, affecting the immune response. Meanwhile, AGTR2 and LIF serve distinct roles in regulating developmental processes and immune responses [90]. After the first day of cell incubation with BGO1, we determined a negative fold change value, which indicated a decrease in the expression of immune-related genes.

**Figure 10:**
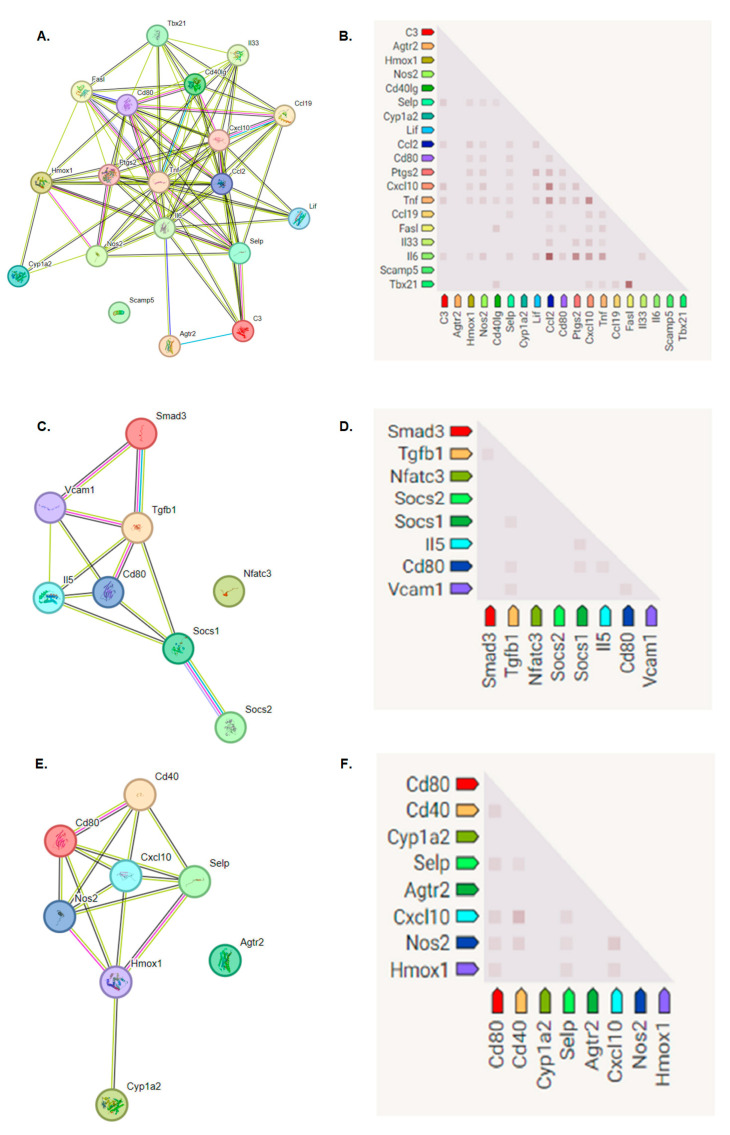
The correlation of proteins between immune response-associated genes. The assessment of immune response-associated genes to acquire protein–protein interaction network diagram was created on the STRING program. (**A**,**C**,**E**) illustrate the protein functional dependence network. Network nodes represent proteins: splice isoforms or post-translational modifications are collapsed, i.e., each node represents all the proteins produced by a single, protein-coding gene locus. Node color: colored nodes represent query proteins and the first shell of interactors. White nodes: the second shell of interactions. Empty nodes: proteins of unknown 3D structure. Filed nodes: some 3D structures are known or predicted. The edges represent protein–protein associations: associations are meant to be specific and meaningful, i.e., proteins jointly contribute to a shared function; this does not necessarily mean they are physically binding to each other. Known interactions: blue—from curated databases; pink—experimentally determined. Predicted interactions: green—gene neighborhood; red—gene fusions; dark blue—gene co-occurrence. Others: yellow—text mining; black—co-expression; violet—protein homology. (**B**,**D**,**F**) illustrate co-expression predicts functional association. In the triangle matrices above, the intensity of color indicates the level of confidence that two proteins are functionally associated, given the overall expression data in the mouse organism.

In turn, after 7 days of cell incubation with the BGO1 biomaterial, we determined a positive fold change value, which indicates an increase in the expression of immune-related genes (see Figure 10B). One of the most significant differences in fold change value was observed for the Socs2 gene, which is responsible for encoding regulatory proteins known as suppressors of cytokine signaling. These proteins play a crucial role in negative feedback loops that regulate cytokine signaling pathways, such as IL-2, IL-3, IL-6 and IL-7 [91]. Upon activation, SOCS2 inhibits further signaling, which results in the dampening of the activation of signaling pathways. Additionally, this protein also impacts the regulation of cell growth and differentiation, which explains the observed increase in cell proliferation over time and the reduced secretion of LDH (see Figure 7 and Figure 8).

Figure 10C,D depicts the functional relationships between proteins exhibiting a significant fold increase in expression on the seventh day of cell incubation with the BGO1 biomaterial. The experiment resulted in notable increases in the expression of the *Cd80* and *Il5* genes. These genes are closely linked to the production of proteins responsible for the activation of T lymphocytes [92,93]. On the other hand, the increased expression of *Socs1* and *Socs2* genes (Figure 10B) can influence the regulation of these genes, helping to counteract inflammation in the targeted tissue, as these proteins directly interact with one another (Figure 10C). Additionally, the elevated expression of genes such as *Smad3* and *Tgfb1* (Figure 10B) has a positive impact on cell proliferation [94], as illustrated in Figure 7, Figure 8 and Figure 10B. This increase in the expression of Smad3 and Tgfb1 genes is likely associated with the presence of graphene, which forms the surface on which the cells are cultured. Other studies, including those by Shim NY et al. [95], Yang Y et al. [96] and Park S et al. [97], have demonstrated that the addition of graphene, a biocompatible material that serves as an excellent substrate for culturing stem cells, influences cell proliferation and Smad3-regulated pathways. Graphene addition also enhances interactions between cells and the extracellular matrix and intercellular junctions through signaling pathways involving TGFB1. These findings offer insights into the observed differences in fold change that determine the increased expression of the *Smad3* and *Tgfb1* genes over time when cells are exposed to BGO1.

The analysis of gene expression changes in cells cultured on the BGO1 biomaterial over time revealed significant positive fold changes and the increased expression of several genes: *Agtr2*, *Cxcl10*, *Cd80*, *Hmox1*, *Cd40*, *Nos2* and *Selp*, as shown in Figure 10C. The expression of these genes significantly influences the activation of the immune system within cells, primarily by stimulating T lymphocytes. In Figure 10E,F, the interrelationships and the final gene expression outcomes are depicted. Most studies on immune responses have historically focused on the cells of the human immune system, where graphene has been shown to impact the activation of specific pathways involved in these processes. For instance, Cebadero-Dominguez et al. [86] investigated the immune response of reductive GO in monocytes and human T lymphocytes. They observed an increase in the levels of IL-6 as early as 4 h after exposing cells to rGO. In our study, an increase in the expression of IL-6 was observed on the seventh day of incubation of L-929 cells (Figure 10A–C). Moreover, a decrease in Tnf expression was observed on the first day of incubation (Figure 10A). This research group also assessed the levels of BAX and BCL-2, which exhibited reduced expression after one day of incubation, which is consistent with our experiments (Figure 10A). Additionally, the researchers determined that the initial day of the incubation of human immune system cells failed to induce a significant release of cytokines, a finding that also aligns with our observations (Figure 10A). Additionally, Yang Z et al. [85] conducted experiments to assess the immunotoxicity of GO on dendritic cells. The results revealed that the expression of selected genes responsible for immune system function, such as Fas, was reduced on the first day of incubation, with diminishing activity observed on the seventh day (Figure 10A,B). It is worth emphasizing that all these genes are known to play crucial roles in immune processes, inflammation and the regulation of homeostasis [49,50,51].

## 4. Conclusions

Recently, there has been a growing interest in the use of nanomaterials in biomedical engineering. Especially, the use of GO as an additive has generated significant attention due to its possible applications in the field of biomedicine. To fully exploit the potential of available nanomaterials in biomedical engineering, it is essential to determine the validity of their use. It usually requires extensive biological and physiochemical testing. The biggest challenge is to develop a biomaterial that is recognized by the immune system as harmless or self-like. In general, biomedical engineering research is focused on the development of a cell-safe biomaterial with significant utility in processing technologies such as 3D bioprinting.

Based on the work carried out, it can be shown that:

1. GO-enriched bioinks are characterized by higher viscosity than the corresponding hydrogels. The storage modulus of the studied bioinks is greater than the loss modulus, indicating that these materials are highly useful in extrusion bioprinting technology.
2. Hydrogels containing GO absorb significantly more water than the bioink, and the greatest water absorption takes place during the first 48 h. The tested materials have significant biodegradability for 21 days.
3. The tested materials show better print resolution and fiber stability compared to the reference sample.
4. The tested materials enriched with GO show significant elasticity of structure.
5. Based on the bioassays, it was concluded that biomaterials with 1% graphene oxide additives do not exhibit cytotoxicity against L-929 cells.
6. In addition, based on the analysis of LDH release and the Alamar Blue assay, it can be concluded that cells cultured on the graphene oxide biomaterial are not damaged; as a result; they do not produce lactate dehydrogenase and show an unimpaired degree of proliferation, except for the culture of cells on the BGO2 biomaterial on the seventh day of the experiment.
7. When analyzing the fold changes in the expression of genes responsible for immune processes, it was found that on the first day of the experiment, there was a decrease in the multiplicity of expression of genes related to the immune system. During the experiment, the expression profile changed. The addition of bioinks with 1% graphene oxide caused an increase in some genes, especially those responsible for the processes of the proliferation and activation of T lymphocytes.
8. The obtained characteristics of the tested materials prove their high utility in 3D bioprinting technology for applications in biotechnology and regenerative medicine.

## Figures and Tables

**Table 1:** Composition of hydrogels and bioinks.

Variant	Concentration [mg/mL]					
	dECM	GO	GelMa	HaMa	LAP	Glycerol
HGO1	-	1	78.3	1.7	1.85	-
HGO2	-	1	130	6.5	1.85	-
BREF	76.6	0	37.5	0.56	1.85	89.8
BGO1	76.6	0.5	37.5	0.56	1.85	89.8
BGO2	76.6	0.5	62	3.1	1.85	89.8

**Table 2:** Swelling ratio and degree of degradation of HGO1, HGO2, BGO1 and BGO2.

Variant	Swelling Ratio [%]	Degree of Degradation [%]	
		Non-Enzymatic	Enzymatic
HGO1	5168	65.97	81.82
HGO2	3329	88.11	92.07
BGO1	607	53.59	78.75
BGO2	513	75.17	90.94

## Data Availability

Data are contained within the article.
